# Antioxidant Interactions between S-allyl-L-cysteine and Polyphenols Using Interaction Index and Isobolographic Analysis

**DOI:** 10.3390/molecules27134089

**Published:** 2022-06-25

**Authors:** Chunming Dong, Guihong Zhao, Lei Tao, Fanghang Qiu, Shujing Wang, Bo Wang, Jian Liu, Shengxia Duan

**Affiliations:** 1 College of Marine and Environmental Sciences, Tianjin University of Science and Technology, Tianjin 300457, China; mingchundongjy@tust.edu.cn; 2 College of Agriculture and Bioengineering, Heze University, Heze 274000, China; sdzgh100@163.com (G.Z.); Taolei00000@163.com (L.T.); qfh1737072105@163.com (F.Q.); 13061346569@163.com (S.W.); caoliwuxi@163.com (B.W.); 3 College of Chemisty and Chemical Engineering, Heze University, Heze 274000, China

**Keywords:** S-allyl-L-cysteine, polyphenols, antioxidant, synergistic effect, interaction index

## Abstract

This work aims to study the antioxidant interactions between S-allyl-L-cysteine (SAC) and six natural polyphenols (quercetin, caffeic acid, sinapic acid, catechin, ferulic acid, and 3,4-dihydroxybenzoic acid) through the measurement of free-radical-scavenging activity of 1,1-diphenyl- 2-picryl-hydrazyl (DPPH), the radical-cation-scavenging activity of 2,2-azino-bis-3-ethylbenzothiazoline-6-sulfonic acid (ABTS), and reducing power. Among the six natural polyphenols, caffeic acid showed the strongest synergistic effect with SAC according to DPPH and reducing power assays. Further investigations based on the results of interaction index and isobologram analysis showed that the antioxidant activity (DPPH, ABTS, and reducing power) of the combination of caffeic acid with SAC presented an increase with the raising of their individual concentrations in their mixture and along with a dose–response manner. The best synergistic effect between caffeic acid and SAC based on DPPH, ABTS, and reducing power assays were observed at the ratio of 1:20, 1:35, and 1:70, respectively. The excellent synergic antioxidant activity of the combination of caffeic acid with SAC in our study suggests SAC has a more broad and effective application prospects in food field.

## 1. Introduction

Garlic (*Allium sativum* L.), a member of the genus Allium, has been widely cultivated and consumed throughout the world as a vegetable condiment and medicinal herb [1]. As a general rule, sulfur compounds are the major bioactive substances of garlic, which endow the garlic with various physiological properties, including antibacterial, antioxidant, anti-inflammatory, and preventive properties against cardiovascular diseases, etc. [2,3,4]. Recently, much attention has been given to S-allyl-L-cysteine (SAC) because of its important roles in the biological functions of black garlic and aged garlic [5,6]. SAC is not only one of the water-soluble cysteine derivatives in garlic, but also an important precursor to the synthesis of alliin and is recognized as the main flavor substance in garlic [7]. In particular, SAC demonstrates an ideal antioxidant activity, scavenging DPPH free radicals, scavenging superoxide radicals, and reducing the human body’s oxidative stress [8,9,10]. On these grounds, further investigations of SAC antioxidant capacity will be beneficial for its potentially wider applications in food areas or other relevant fields.

In previous years, polyphenols in a large number of plants and foods, presenting significant physiological activities, have been investigated in depth [11,12,13]. The chief reason that polyphenols have received such attention can be ascribed to their advanced antioxidant performance compared to other antioxidants. Together with the high content of polyphenols in the human diet (approximately one-third), polyphenols probably play a pivotal role in preventing certain oxidation-associated diseases, such as diabetes, cancer, and hypertension [14]. According to previous literature, the antioxidant mechanisms of polyphenols mainly include an eliminating effect directly against free radicals, efficient chelating of trace-metal ions, inhibition of oxidizing enzymes, and regeneration of certain antioxidants [15]. Moreover, polyphenols do not act alone and they can actually function as co-antioxidants.

Recently, studying the interactions between antioxidants has been a research trend for the discovery of more effective antioxidant combinations. It is generally agreed that three different types of interactions exist between antioxidants, including synergism, antagonism, and additive effect [16]. However, interactions between antioxidants are frequently reported, for example, Schroeder et al. [17] demonstrated that the interaction between α-tocopherol and β-carotene changed from synergism to antagonism if the mixing concentration of α-tocopherol was close to β-carotene. Guo et al. [18] proved that the antioxidant effect of γ-terpinene could be achieved by reducing quinone compounds to catechols, and synergistic antioxidation of γ-terpinene and monophenol mixtures was different with γ-terpinene and polyphenol mixtures from the perspective of mechanism. Noguer et al. [19] found that ascorbic acid could mix with 3,4-dihydroxyphenylacetic acid, and their combination showed a good antioxidant effect. However, investigations on antioxidant interaction of SAC and natural polyphenols have not been reported so far, necessitating further research.

The goal of this study was to research the antioxidant interactions between SAC and six natural polyphenols (quercetin, caffeic acid, sinapic acid, catechin, ferulic acid, and 3,4-dihydroxybenzoic acid) by the radical-scavenging activity of DPPH, ABTS, and reducing power. Moreover, different concentrations and ratios of SAC and caffeic acid on the influence of their mixture was further studied by interaction index and isobolographic analysis in order to discover their optimal combination. Our study would provide a theoretical basis for the development of antioxidant products of SAC and its combined natural antioxidants.

## 2. Results and Discussion

### 2.1. Antioxidant Activity of Individual Compounds including SAC and Six Polyphenols

The three antioxidant activity assays in our study included DPPH, ABTS, and reducing power assays, which belong to electron transfer assays [20,21,22]. However, in DPPH and ABTS assays, factors such as light, oxygen, and pH easily influence the color of the reactant’s mixture. On the other hand, the reducing power approach is accompanied by uncertainties related to the reaction mechanism: there is a discrepancy between the measured value and the ability of antioxidant to interact with the free radicals generated by the metal ions or to bind to the metal ions themselves. Nevertheless, the described drawbacks among these assays do not prevent their wide application. Hence, the antioxidant activity of different individual compounds or their mixtures in this study were estimated by DPPH, ABTS, and reducing power assays due to their highly sensitive assays with reproducible results and the corresponding assays results were listed in Table 1 [23].

**Figure 1:**
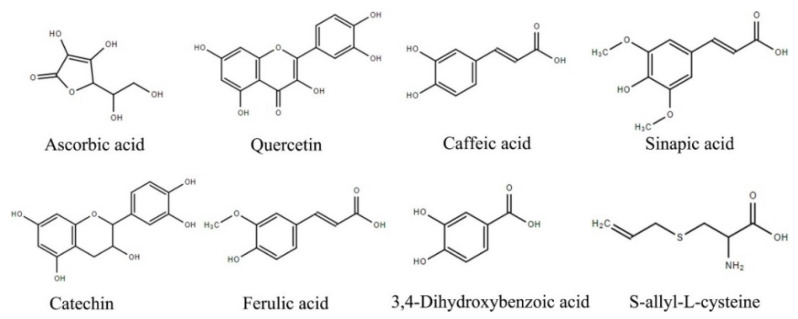
Chemical structure of 6 polyphenols and SAC.

The EC_50_ values of different individual compounds in the DPPH assay were reduced (*p* < 0.05) in the order: SAC > caffeic acid, ascorbic acid, ferulic acid, sinapic acid, 3,4-dihydroxybenzoic acid, catechin, and quercetin. The EC_50_ values of different individual compounds in the ABTS assay were decreased (*p* < 0.05) in the order: SAC > ascorbic acid > caffeic acid > sinapic acid, 3,4-dihydroxybenzoic acid > catechin > ferulic acid, quercetin. The OD_0.5_ values of different individual compounds in the reducing power assay were decreased (*p* < 0.05) in the order as follows: SAC > sinapic acid, quercetin, catechin, ascorbic acid, ferulic acid, 3,4-dihydroxybenzoic acid, caffeic acid. The EC_50_ and OD_0.5_ values of six polyphenols were all much lower than that of SAC (*p* < 0.05), which presented the lowest antioxidant capacity in these assays. When compared with ascorbic acid, the antioxidant activity of SAC was from 30 to 66 times weaker. Moreover, different antioxidant activities of the six natural polyphenols (quercetin, caffeic acid, sinapic acid, catechin, ferulic acid, 3,4-dihydroxybenzoic acid) were also confirmed in our study, which varied depending upon their structure and the mechanism of the antioxidant assay. According to previous literature [24], the antioxidant capacity of polyphenols can be affected by a number of factors, including the oxidation reaction system, the compound’s hydrophobic property, and the group of donating electrons, etc. Among these, the number and position of hydroxyl groups are particular factors contributing to the antioxidant capacity of polyphenols. Particularly, the substitution of hydroxyl group at o-diphenol is considered as more important than other positions, and the number of phenolic hydroxyl groups is not a case of the more the better. For example, the hydroxyl group of ortho phenolic on catechin B ring (Figure 1) plays the most important role in ABTS radical cation scavenging ability, while the contribution of hydroxyl group of ortho phenolic on A ring and C ring is very small [25].

Further, in our study, ferulic acid and sinapic acid, whose phenolic hydroxyl ortho is methoxy, have stronger ABTS radical cation scavenging abilities than caffeic acid (*p* < 0.05), suggesting the methoxy group of styrenic acid polyphenols contributes more to free radical scavenging than its phenolic hydroxyl group. Furthermore, the reducing power of caffeic acid was stronger than sinapic acid (*p* < 0.05), which is not only related to the o-diphenol hydroxyl group, but also attributed to the existence of -CHCHCOO- side chain. The conjugated double bond on the side chain can also increase the charge distribution of the phenoxy radical through resonance and produce a strong electron pushing ability, so as to enhance the reduction ability of phenolic acid. In all, these results clearly indicated that the antioxidant effect (DPPH, ABTS, and reducing power) of six polyphenols were significantly higher than that of SAC (*p* < 0.05). The principal reason for the lower antioxidant effect of SAC can be attributed to a lesser number of phenolic hydroxyl groups compared with that of the six polyphenols. Thus, studies on antioxidant interactions of the combination between SAC and individual polyphenols are of great significance and value, which is beneficial for the antioxidant activity increase of SAC.

### 2.2. Antioxidant Interactions of SAC and Six Polyphenols

Based on the antioxidant activity result of different individual compounds, SAC, and six polyphenols at theoretical concentration with equivalent effect (5%, 10%, 20%, 30%, 35%, 40%, 45% of DPPH and ABTS radical scavenging activity, 0.05, 0.10, 0.20, 0.30, 0.35, 0.40, 0.45 absorption values of reducing power) were used to prepare the binary mixtures, then evaluated their median inhibitory activities (EC_50_ or OD_0.5_ values) and interaction indexes (γ) for each assay and each combination. The results were presented in Table 2.

A synergistic effect can be implied by interaction index values < 1. A value > 1 suggests an antagonistic effect, while equaling to 1 indicates an additional interaction. According to the interaction indexes (γ) in Table 2, the combination of SAC and six polyphenols all showed synergistic effects in DPPH radical scavenging, and the order of synergism from weak to strong (*p* < 0.05) is: ferulic acid + SAC, sinapic acid + SAC, quercetin + SAC < catechin + SAC, 3,4-dihydroxybenzoic acid + SAC < caffeic acid + SAC. This showed that the synergistic effect of caffeic acid and SAC was the strongest (*p* < 0.05). In particular, antagonistic effects of all combinations of SAC and the six polyphenols were observed in ABTS radical cation scavenging. Among these mixtures, ferulic acid + SAC and sinapic acid + SAC showed stronger antagonistic effects (*p* < 0.05) than 3,4-dihydroxybenzoic acid + SAC and quercetin + SAC. Furthermore, mixtures of quercetin + SAC, caffeic acid + SAC, sinapic acid + SAC, catechin + SAC showed synergistic effects in the reducing power assay, while caffeic acid + SAC showed a stronger synergistic effect (*p* < 0.05) than catechin + SAC, sinapic acid + SAC and quercetin + SAC. Mixtures of ferulic acid + SAC and 3,4-dihydroxybenzoic acid + SAC showed an antagonistic effect. Up to now, the synergistic mechanism of antioxidation has mainly included five categories: repair and regeneration, coupling oxidation, absorption of oxygen, change of enzyme activity and complexation of metal ions [26]. However, not all antioxidants show a good synergistic effect with other compounds. There are also some influencing factors, such as reduction potential, relative concentration, and reaction system [27]. Furthermore, the antagonism between antioxidants may be affected by the formation of antioxidant free-radical adducts or the competition between antioxidant regeneration, and the microenvironment of one antioxidant is changed by another antioxidant [28,29,30]. In most cases, the synergy reaches the highest level in a specific proportion. However, caffeic acid showed the strongest synergistic effect with SAC according to DPPH and reducing power assays.

### 2.3. Antioxidant Interactions of SAC and Caffeic Acid at Different Proportions

#### 2.3.1. Antioxidant Activity of Caffeic Acid, VC and SAC at Different Concentrations

**Figure 2:**
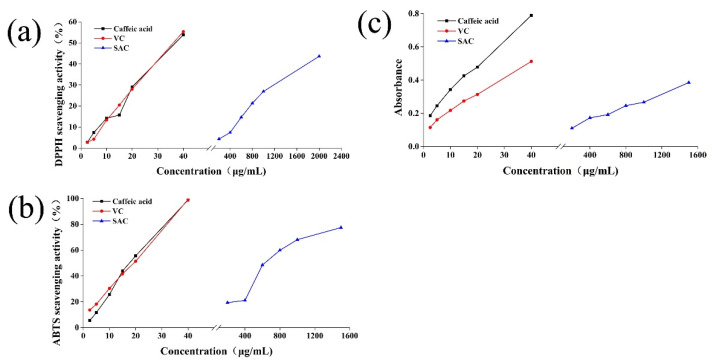
Antioxidant activity of caffeic acid, VC, and SAC at different concentrations. (**a**) 1,1-diphenyl-2-picrylhydrazyl (DPPH) free-radical-scavenging activity, (**b**) 2,2′-azino-bis-3-ethylbenzthiazoline-6-sulphonic acid (ABTS) radical-cation-scavenging activity, (**c**) reducing power.

Figure 2 showed the free-radical-scavenging activity (DPPH), radical-cation-scavenging activity (ABTS) and reducing power of SAC, caffeic acid, and ascorbic acid (VC) at different concentrations. It can be seen that the antioxidant activity–concentration (DPPH, ABTS, and reducing power) curve of caffeic acid is close to that of VC in the figure, and antioxidant activity–concentration (DPPH, ABTS, and reducing power) curve of SAC is quite far away from caffeic acid and VC, which was consistent with the previous result in Table 1, determining the notable lower antioxidant activity (DPPH, ABTS, and reducing power) of SAC than caffeic acid and VC (*p* < 0.05). In short, the antioxidant activity of SAC, VC and caffeic acid was more intuitively reflected by Figure 2.

#### 2.3.2. Antioxidant Activity for the Mixture of Caffeic Acid and SAC

A weak antioxidant could be mixed with a strong antioxidant, while the overall radical-scavenging capacity of the mixture may be enhanced through the regeneration of a strong antioxidant from a weak antioxidant [31]. To better understand the participation of the single substances in a mixture, different proportions of SAC and caffeic acid were further investigated in different assays (DPPH, ABTS, and reducing power assays) (Table 3).

Additionally, the proportions (different concentrations of caffeic acid and SAC under the same caffeic acid to SAC ratio) of SAC and caffeic acid were selected on the basis of EC_50_ or OD_50_ values measured by DPPH, ABTS, and reducing power assays. From Table 3, antioxidant capacities of mixtures SAC and caffeic acid were all increased, with their single substance increasing concentration (SAC and caffeic acid) for the same concentration ratio.

#### 2.3.3. Interaction Index Values for the Mixture of Caffeic Acid and SAC

It can be seen from Table 4 (DPPH scavenging activity) that the EC_50mix_ values of mixture caffeic acid + SAC corresponding to the ratio 1:10, 1:15, 1:20, and 1:25 were 264.02, 291.46, 314.31 and 430.55 μg/mL, respectively.

Therefore, the mix ratios of caffeic acid and SAC for scavenging DPPH free radicals were decreased (*p* < 0.05) in the order as 1:10 > 1:15 > 1:20 > 1:25. Moreover, according to the interaction index (γ), caffeic acid, and SAC at the four mix ratios all showed synergistic effects (γ < 1) and the order of synergistic effects were as follows (*p* < 0.05): 1:20 > 1:25 > 1:10. To further illustrate the synergistic effect between caffeic acid and SAC, the synergistic rate of caffeic acid and SAC was calculated according to the equation of synergistic rate/% = (EC_50add_ − EC_50mix_)/EC_50add_ [32]. In this part, the synergistic rate of caffeic acid and SAC was consistent with the result of the interaction index. For example, the mixture of caffeic acid and SAC at 1:20 ratio possessed the lowest interaction index (0.51, *p* < 0.05) compared with that obtained at ratios of 1:25 and 1:10 and the highest synergistic rate (49.39%). Thus, both synergistic rate and interaction index indicated that 1:20 was the appropriate ratio with the best synergistic effect between caffeic acid and SAC based on DPPH assay.

On the other hand, it can be seen from Table 4 (ABTS radical cation scavenging activity) that the EC_50mix_ values of mixture caffeic acid and SAC corresponding to the ratio 1:30, 1:35, 1:40, and 1:45 were 371.16, 269.09, 320.58, and 318.65 μg/mL, respectively. Therefore, the mix ratios of caffeic acid and SAC for scavenging ABTS cation radical were decreased (*p* < 0.05) in the order as 1:35 > 1:45 > 1:40 > 1:30. Moreover, according to the interaction index (γ), caffeic acid and SAC at the three mix ratios (1:35, 1:45, 1:40) showed synergistic effects (γ < 1) and one ratio (1:30) presented antagonistic effect (γ > 1). Considering synergistic rate and interaction index, caffeic acid and SAC at a ratio of 1:35 was the ratio with the best synergistic effect based on ABTS assay.

From the reducing powers shown in Table 4, the EC_50mix_ values of mixture caffeic acid and SAC corresponding to the ratio 1:70, 1:75, 1:80, and 1:85 was 495.87, 542.1, 647.05, and 621.35 μg/mL, respectively. And these four ratios of caffeic acid and SAC all presented synergistic effects (γ < 1), consistent with the results of DPPH assay. The mix ratios of caffeic acid and SAC for reducing power were also decreased (*p* < 0.05) in the order 1:70 > 1:75 > 1:85 > 1:80. Combining the results of synergistic rate and interaction index, caffeic acid and SAC at 1:70 ratio was the ratio with best synergistic effect based on reducing power.

In addition, EC_50mix_ was compared with EC_50add_ for each mixture of caffeic acid and SAC by independent *t*-test in DPPH, ABTS, and reducing power assays. A synergistic interaction between the mixtures was determined if the mixture presented a notable lower EC_50mix_ than EC_50add_. Except the caffeic acid and SAC at ratio 1:30, the other mixtures of caffeic acid and SAC all have a significant lower EC_50mix_ value than EC_50add_ value, suggesting that combination of caffeic acid and SAC at an appropriate proportion could obtain a mixture with a good antioxidative function.

#### 2.3.4. Isobolographic Analysis of the Mixture of Caffeic Acid and SAC

**Figure 3:**
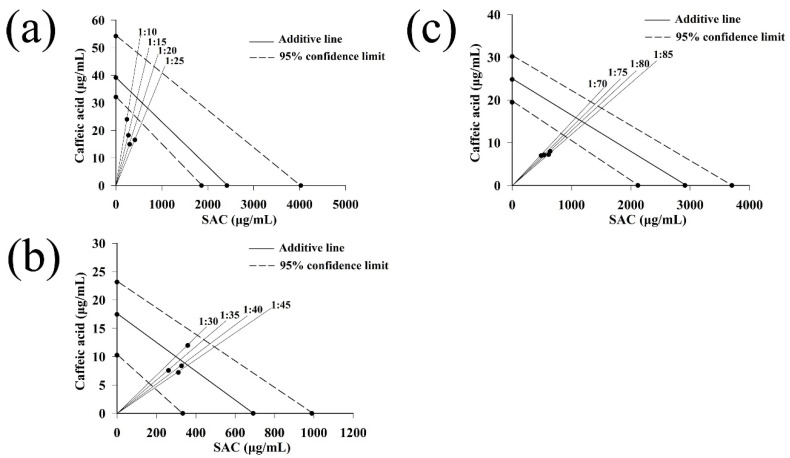
Isobologram for the mixture of caffeic acid and SAC in the DPPH, ABTS, and reducing power assays. (**a**) DPPH assay, (**b**) ABTS assay, (**c**) reducing power assay.

In order to make a scientific visual evaluation of the antioxidant interaction, isobolographic analysis has often been adopted to graphically display a concise and straightforward interaction [33]. Therefore, isobolographic analysis was used to study interactions depending on the mixing ratio of caffeic acid and SAC (Figure 3). The individual EC_50_ values for caffeic acid and SAC were plotted on the *x*- and *y*-axes, respectively. The two points of EC_50_ values were linked and the connection line was a theoretical additive line (isobola). The points lying above and below isobola were acknowledged as antagonism and synergism between mixed compounds, respectively [23]. The obtained isobolograms on Figure 3 confirmed synergistic effects between caffeic acid and SAC at ratios 1:20, 1:15, 1:25, 1:10 in DPPH assay, 1:35, 1:45, 1:40 in ABTS assay, and 1:70, 1:75, 1:80, and 1:85 in reducing power assay. The exception was the 1:30 ratio of caffeic acid and SAC in the ABTS assay, which point lying above isobola. In all, the isobolographic results were united with the interaction index.

### 2.4. Absorbance Change of the Mixture of Caffeic Acid and SAC with Time

**Figure 4:**
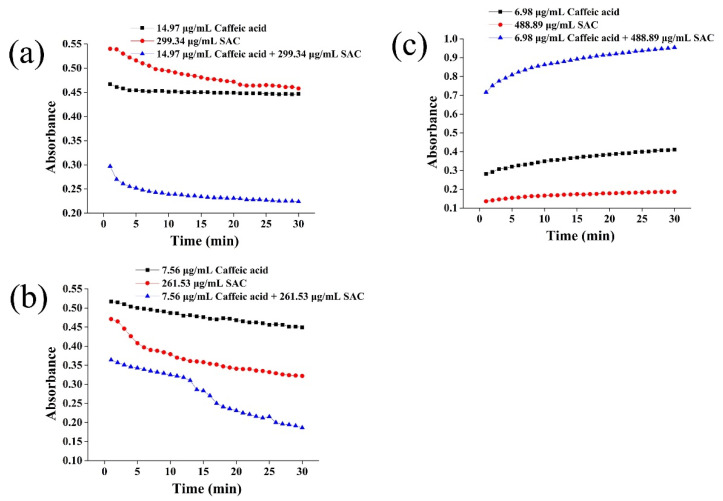
The kinetic curves of scavenged DPPH free radicals, ABTS radical cations, and reducing power by caffeic acid, SAC, and their mixture. (**a**) DPPH assay, (**b**) ABTS assay, (**c**) reducing power assay.

In order to explore the synergistic mechanism of the absorbance changes of the mixture of caffeic and SAC with time in DPPH, the ABTS and reducing power experiments were further investigated (Figure 4). It can be seen from Figure 4 that there existed a significant difference in the absorbance of SAC before and after compounding with caffeic acid. In the DPPH assay, a rapid decrease of the absorbance of mixture caffeic acid and SAC was observed at the first 5 min. This rapid decrease at the initial time may be attributed to the electrons of a phenol molecule or its phenoxide anion shifting to DPPH free radical, and the following decay was resulted from the remaining activity of oxidation reactants [34]. In the ABTS assay, the absorbance of mixture caffeic acid + SAC kept a decrease continually from reaction starting time to 25 min, and tended to be flatter after 25 min. In the reducing power assay, the mixture absorbance changed greatly in the first 10 min, and tended to be flat after 20 min. This inconsistency may be related to the different mechanisms of the three antioxidant reactions. DPPH and ABTS are both decolorization assays; for DPPH, radicals should be transferred to stable diamagnetic molecules through the reactions with antioxidants [35], nevertheless, the ABTS assay is conducted via the reaction between antioxidants and ABTS radical cation. The potassium ferricyanide reducing power assay is evaluated via the reaction of reducing ferric complex to the ferrous form by antioxidants, the absorbance of which would be increased. However, it is worth noting that the absorbance of the mixture of caffeic acid and SAC was obviously different with SAC or caffeic acid alone, especially in the DPPH and reducing power experiments, which also indirectly proves the two antioxidants have a synergistic effect.

## 3. Materials and Methods

### 3.1. Chemicals and Reagents

Six natural polyphenols (quercetin, caffeic acid, sinapic acid, catechin, ferulic acid, 3,4-dihydroxybenzoic acid), ascorbic acid, HPLC-grade methanol, as well as potassium persulfate, trichloroacetic acid, ferric chloride, and potassium ferricyanide were obtained from Shanghai Macklin Biochemical Co., Ltd. (Shanghai, China). While DPPH, ABTS, and SAC were supplied by Shanghai yuanye Bio-Technology Co., Ltd. (Shanghai, China).

### 3.2. Measurement of Scavenging Activity of DPPH, ABTS, and Reducing Power

The determination of 1,1-diphenyl-2-picrylhydrazyl (DPPH) free-radical-scavenging ability was conducted as previously proposed by the method of Jia et al. [36]. First, DPPH solution (0.2 mM) was prepared with 70% ethanol/water solution as solvent, then the solution of DPPH (2.0 mL) and sample (2.0 mL) was evenly mixed and reacted for 30 min without light (25 ± 2 °C). With a control of ascorbic acid (VC), the absorbance value of reactant (A_i_) was tested at 517 nm. The antioxidant activity of DPPH free-radical-scavenging ability was expressed using EC_50_ value, i.e., the concentration (µg/mL) demanded to scavenge 50% of DPPH radicals calculated by the nonlinear regression or curve fit versus the corresponding to the sample concentration using the SPSS software (Version 22.0, Chicago, IL, USA). In addition, the displayed Formula (1) below was adopted to estimate the DPPH scavenging rate:

Scavenging rate% = 1 − (A_1_ − A_0_)/A_1_ × 100 (1)

where, A_1_ suggests the mixture absorbance of DPPH and 70% ethanol/water solution with the equal volume (2.0 mL), and A_0_ indicates the solution absorbance of sample and DPPH with the same volume (2.0 mL).

The determination of ABTS radical-cation-scavenging capacity was done according to a previous study [37]. In short, 0.192 g ABTS and 0.033 g potassium persulfatein was dissolved in 50 mL distilled water, respectively, mixed evenly, and placed in the dark at room temperature for 12~16 h. Then, a certain amount of distilled water was added to the mother solution, and the solution was diluted to the absorbance value of 0.7 ± 0.02 at 734 nm, which was used as the ABTS working solution of this experiment. Then, 0.5 mL Caffeic acid, SAC or composite solution of different concentrations was added into the test tube, and 5 mL ABTS working solution added, respectively, and mixed evenly (added 0.5 mL of 70% methanol solution in the control tube and 0.5 mL of distilled water in the blank tube). Placing it at room temperature in a dark place for 10 min, the absorbance value was measured at 734 nm, and the test repeated three times. The antioxidant activity of ABTS radical-cation-scavenging ability was also expressed by EC_50_ value and evaluated by using the following Equation (2):

Scavenge rate % = (A_1_ − A_0_)/A_1_ × 100 (2)

The absorbance of the control (containing all reagents except the sample) is presented by A_0_, and the absorbance of the sample is showed via A_1_.

The potassium ferricyanide reducing power was measured as indicated previously [38]. Adding 2.5 mL of sample solution (polyphenol, SAC, or composite solution) with 2.5 mL of distilled water and 2.5 mL 1% (*w*/*v*) potassium ferricyanide solution into the test tube, fully mixed and incubated at 50 °C for 20 min, then 2.5 mL 10% (*w*/*v*) trichloroacetic acid solution was added, mixed sufficiently and left to stand for 10 min. Taking the 2.5 mL mixed solution in a new test tube, 2.5 mL distilled water and 0.5 mL 1% (*w*/*v*) ferric chloride solution was then added, measuring its absorbance at 700 nm, and the determination repeated three times. The effective concentration required for the absorbance to reach 0.5 is defined as the OD_0.5_ value. The smaller the value of OD_0.5_, the stronger the reduction ability of the sample.

### 3.3. Assessment of the Antioxidant Interactions between SAC and Polyphenols

Interactions between SAC and polyphenols were first reflected by the interaction index (γ) [39]. The interaction index (γ) was valued by the following Equation (3):

γ = EC_50Amix_/EC_50A_ + EC_50Bmix_/EC_50B_ (3)

where EC_50A_ and EC_50B_ represent the EC_50_ values of antioxidants A and B in their individual solution, respectively, while EC_50Amix_ and EC_50Bmix_ indicate the EC_50_ values of antioxidants A and B in their combined solution respectively.

Besides, interactions between SAC and polyphenols were also evaluated using isobolographic analysis at the EC_50_ level of the effect, as described previously [39,40]. Briefly, for the antioxidant mixture, experimental EC_50mix_ was calculated by adding EC_50Amix_ and EC_50Bmix_ together, which was further compared to a theoretical additive EC_50add_. EC_50add_ was calculated by the following Equation (4):

EC_50add_ = EC_50A_/(P_A_ + RP_B_) (4)

where R is the potency ratio (EC_50A_/EC_50B_), P_A_ and P_B_ represent the proportion of antioxidants A and B in their mixture.

### 3.4. Statistical Analysis

All experiments were executed in triplicate and performed as mean ± standard deviation (n = 3). SPSS software (Version 22.0, Chicago, IL, USA) was applied to analyze the data. Isobolograms for SAC and caffeic acid in the antioxidant assay were plotted by SigmaPlot (Version 12.0, San Jose, CA, USA).

## 4. Conclusions

Interaction indexes and isobologram analysis were used to study the synergistic antioxidant effects of SAC and six natural polyphenols (quercetin, caffeic acid, sinapic acid, catechin, ferulic acid, and 3,4-dihydroxybenzoic acid) by detecting the scavenging activity of DPPH, ABTS, and reducing power. A much lower antioxidant effect (DPPH, ABTS and reducing power) of SAC was found when compared with the six polyphenols (*p* < 0.05). Among these six natural polyphenols, caffeic acid showed the strongest synergistic effect (*p* < 0.05) with SAC according to the results of DPPH and reducing power assays. Moreover, the best synergistic antioxidant between caffeic acid and SAC based on DPPH, ABTS, and reducing power assays were observed at the ratio of 1:20, 1:35, and 1:70, respectively. Additionally, the method of mixing SAC with polyphenols could obtain a mixed antioxidant with excellent antioxidation property, and indicates a more broad and effective application prospects of SAC in food field.

## Figures and Tables

**Table 1:** EC_50_ and OD_50_ values of individual compounds for DPPH, ABTS, and reducing power assays.

Index	EC_50_ of DPPH Scavenging Activity/(μg/mL)	EC_50_ of ABTS Scavenging Activity/(μg/mL)	OD_0.5_ of Reducing Power/(μg/mL)
Ascorbic acid	36.39 ± 0.2 ^a^	23.08 ± 0.16 ^a^	48.83 ± 0.15 ^ab^
Quercetin	8.39 ± 0.09 ^b^	5.10 ± 0.03 ^b^	69.08 ± 1.02 ^bc^
Caffeic acid	39.16 ± 0.45 ^a^	17.45 ± 0.03 ^c^	24.85 ± 0.48 ^a^
Sinapic acid	17.11 ± 0.17 ^ab^	12.40 ± 0.11 ^d^	101.26 ± 3.98 ^c^
Catechin	10.26 ± 0.33 ^ab^	9.41 ± 0.27 ^e^	61.92 ± 0.54 ^abc^
Ferulic acid	21.76 ± 0.42 ^ab^	5.11 ± 0.02 ^b^	46.31 ± 0.74 ^ab^
3,4-Dihydroxybenzoic acid	11.39 ± 0.27 ^ab^	12.21 ± 0.17 ^d^	29.05 ± 0.19 ^ab^
SAC	2416.09 ± 31.10 ^c^	691.86 ± 6.03 ^f^	2864.63 ± 65.06 ^d^

Note: The letters indicate significant differences determined by Duncan’s multiple range tests (*p* < 0.05).

**Table 2:** Interaction index values for the combination of the different compounds determined by DPPH, ABTS, and reducing power.

	Compound Combination	EC_50A_	EC_50Amix_	EC_50B_	EC_50Bmix_	Interaction Index (γ)
DPPH free radical scavenging activity	Quercetin + SAC	8.39 ± 0.09	4.05 ± 0.04	2416.09 ± 31.10	507.12 ± 11.6	0.69 ± 0.03 ^a^
	Caffeic acid + SAC	39.16 ± 0.45	12.01 ± 0.06	2416.09 ± 31.10	305.43 ± 4.04	0.43 ± 0.04 ^b^
	Sinapic acid + SAC	17.11 ± 0.17	6.49 ± 0.12	2416.09 ± 31.10	917.01 ± 16.81	0.76 ± 0.05 ^ad^
	Catechin + SAC	10.26 ± 0.33	3.21 ± 0.04	2416.09 ± 31.10	673.62 ± 9.9	0.59 ± 0.02 ^c^
	Ferulic acid + SAC	21.76 ± 0.42	9.45 ± 0.09	2416.09 ± 31.10	831.96 ± 9.55	0.78 ± 0.06 ^d^
	3,4-Dihydroxybenzoic acid + SAC	11.39 ± 0.27	4.29 ± 0.11	2416.09 ± 31.10	409.66 ± 19.4	0.55 ± 0.03 ^c^
ABTS radical cation scavenging activity	Quercetin + SAC	5.10 ± 0.03	3.47 ± 0.08	691.86 ± 6.03	401.31 ± 13.01	1.26 ± 0.05 ^a^
	Caffeic acid + SAC	17.45 ± 0.03	12.63 ± 0.02	691.86 ± 6.03	351.97 ± 3.04	1.23 ± 0.07 ^ab^
	Sinapic acid + SAC	12.40 ± 0.11	9.59 ± 0.14	691.86 ± 6.03	417.64 ± 12.78	1.38 ± 0.05 ^c^
	Catechin + SAC	9.41 ± 0.27	7.02 ± 0.04	691.86 ± 6.03	379.07 ± 8.56	1.29 ± 0.08 ^ac^
	Ferulic acid + SAC	5.11 ± 0.02	3.85 ± 0.06	691.86 ± 6.03	443.47 ± 13.66	1.39 ± 0.06 ^c^
	3,4-Dihydroxybenzoic acid + SAC	12.21 ± 0.17	8.07 ± 0.02	691.86 ± 6.03	335.65 ± 1.32	1.15 ± 0.03 ^b^
Reducing power	Quercetin + SAC	69.08 ± 1.02	17.15 ± 0.14	2864.63 ± 65.06	1419.26 ± 1.44	0.74 ± 0.05 ^a^
	Caffeic acid + SAC	24.85 ± 0.48	5.71 ± 0.41	2864.63 ± 65.06	1049.42 ± 21.45	0.60 ± 0.01 ^c^
	Sinapic acid + SAC	101.26 ± 3.98	21.74 ± 0.19	2864.63 ± 65.06	1646.79 ± 4.04	0.79 ± 0.02 ^ab^
	Catechin + SAC	61.92 ± 0.54	21.97 ± 0.11	2864.63 ± 65.06	1431.57 ± 4.8	0.85 ± 0.03 ^b^
	Ferulic acid + SAC	46.31 ± 0.74	31.36 ± 0.14	2864.63 ± 65.06	2014.44 ± 2.7	1.38 ± 0.04 ^d^
	3,4-Dihydroxybenzoic acid + SAC	29.05 ± 0.19	15.20 ± 0.11	2864.63 ± 65.06	1534.46 ± 2.73	1.06 ± 0.05 ^e^

Note: EC_50A_ and EC_50B_ represent the EC_50_ values of antioxidants A and B in their individual solution respectively, while EC_50Amix_ and EC_50Bmix_ indicate the EC_50_ values of antioxidants A and B in their combined solution, respectively. It should be stated that the EC_50_ values are actually OD_0.5_ values in the part of reducing power in order to facilitate the presentation of table. The letters indicate significant differences determined by Duncan’s multiple range tests (*p* < 0.05).

**Table 3:** Antioxidant activity for the mixture of caffeic acid and SAC determined by DPPH, ABTS, and reducing power assays.

Caffeic Acid: SAC (Concentration Ratio)	DPPH Assay			Caffeic Acid: SAC (Concentration Ratio)	ABTS Assay			Caffeic Acid: SAC (Concentration Ratio)	Reducing Power Assay		
	Concentration of Caffeic Acid (µg/mL)	Concentration of SAC (µg/mL)	DPPH Free Radical Scavenging Activity (%)		Concentration of Caffeic Acid (µg/mL)	Concentration of SAC (µg/mL)	ABTS Radical Cation Scavenging Activity (%)		Concentration of Caffeic Acid (µg/mL)	Concentration of SAC (µg/mL)	Absorption
1:10	20	200	48.05 ± 0.31 ^a^	1:30	20	600	99.25 ± 0.54 ^a^	1:70	15	1050	0.87 ± 0.001 ^a^
	16	160	37.87 ± 0.46 ^b^		16	480	97.09 ± 0.17 ^b^		13	910	0.81 ± 0.001 ^b^
	14	140	33.06 ± 0.11 ^c^		14	420	85.93 ± 0.20 ^c^		10	700	0.77 ± 0.001 ^c^
	12	120	27.65 ± 0.28 ^d^		12	360	74.54 ± 0.29 ^d^		7	490	0.56 ± 0.001 ^d^
	10	100	24.22 ± 0.46 ^e^		10	300	66.86 ± 0.17 ^e^		5	350	0.48 ± 0.001 ^e^
	8	80	18.88 ± 0.22 ^f^		8	240	52.51 ± 0.32 ^f^		2	140	0.23 ± 0.002 ^f^
1:15	20	300	58.11 ± 0.17 ^a^	1:35	20	700	96.91 ± 0.34 ^a^	1:75	15	1125	1.05 ± 0.002 ^a^
	16	240	45.82 ± 0.47 ^b^		16	560	96.60 ± 0.37 ^a^		13	975	0.91 ± 0.001 ^b^
	14	210	38.91 ± 0.21 ^c^		14	490	92.52 ± 0.37 ^b^		10	750	0.78 ± 0.002 ^c^
	12	180	31.35 ± 0.32 ^d^		12	420	83.88 ± 0.25 ^c^		7	525	0.56 ± 0.002 ^d^
	10	150	24.34 ± 0.32 ^e^		10	350	72.76 ± 0.35 ^d^		5	375	0.48 ± 0.001 ^e^
	8	120	16.75 ± 0.62 ^f^		8	280	55.67 ± 0.37 ^e^		2	150	0.24 ± 0.001 ^f^
1:20	20	400	69.98 ± 0.36 ^a^	1:40	20	800	98.82 ± 0.25 ^a^	1:80	15	1200	0.81 ± 0.002 ^a^
	16	320	55.86 ± 0.31 ^b^		16	640	98.05 ± 0.20 ^b^		13	1040	0.80 ± 0.003 ^b^
	14	280	51.69 ± 0.25 ^c^		14	560	91.69 ± 0.18 ^c^		10	800	0.68 ± 0.003 ^c^
	12	240	44.17 ± 0.22 ^d^		12	480	90.61 ± 0.16 ^d^		7	560	0.52 ± 0.001 ^d^
	10	200	32.75 ± 0.32 ^e^		10	400	77.31 ± 0.32 ^e^		5	400	0.43 ± 0.001 ^e^
	8	160	25.20 ± 0.43 ^f^		8	320	63.42 ± 0.36 ^f^		2	160	0.22 ± 0.001 ^f^
1:25	20	500	71.65 ± 0.32 ^a^	1:45	20	900	98.57 ± 0.36 ^a^	1:85	15	1275	1.01 ± 0.008 ^a^
	16	400	52.25 ± 0.44 ^b^		16	720	98.16 ± 0.20 ^ab^		13	1105	0.94 ± 0.001 ^b^
	14	350	45.38 ± 0.28 ^c^		14	630	97.82 ± 0.18 ^b^		10	850	0.76 ± 0.001 ^c^
	12	300	41.78 ± 0.44 ^d^		12	540	95.6 ± 0.32 ^c^		7	595	0.55 ± 0.001 ^d^
	10	250	30.95 ± 0.61 ^e^		10	450	77.27 ± 0.23 ^d^		5	425	0.46 ± 0.003 ^e^
	8	200	21.90 ± 0.55 ^f^		8	360	66.29 ± 0.17 ^e^		2	170	0.22 ± 0.001 ^f^

Note: The letters indicate significant differences determined by Duncan’s multiple range tests (*p* < 0.05).

**Table 4:** Interaction index values for the mixture of caffeic acid and SAC determined by DPPH, ABTS, and reducing power assays.

	Concentration Ratio	EC_50Amix_	EC_50Bmix_	EC_50 add_	EC_50 mix_	Synergistic Rate/%	Interaction Index (γ)
DPPH free radical scavenging activity	1:10	24.01 ± 0.09	240.01 ± 0.92	370.66 ± 4.29	264.02 ± 1.02 ** ^a^	28.77 ± 0.66 ^a^	0.71 ± 0.01 ^a^
	1:15	18.22 ± 0.30	273.24 ± 4.50	503.99 ± 5.87	291.46 ± 4.80 ** ^b^	42.16 ± 1.52 ^b^	0.58 ± 0.02 ^bc^
	1:20	14.97 ± 0.08	299.34 ± 1.52	621.01 ± 7.28	314.31 ± 1.60 ** ^c^	49.39 ± 0.34 ^c^	0.51 ± 0.03 ^c^
	1:25	16.56 ± 0.02	413.99 ± 0.46	724.53 ± 8.54	430.55 ± 0.48 ** ^d^	40.57 ± 0.74 ^b^	0.59 ± 0.07 ^b^
ABTS radical cation scavenging activity	1:30	11.97 ± 0.21	359.18 ± 6.21	307.96 ± 1.42	371.16 ± 6.42 ** ^a^	-20.53 ± 2.62 ^a^	1.21 ± 0.03 ^a^
	1:35	7.56 ± 0.15	261.53 ± 0.10	333.68 ± 1.63	269.09 ± 0.13 ** ^b^	19.36 ± 0.42 ^b^	0.81 ± 0.01 ^b^
	1:40	8.08 ± 0.30	327.61 ± 16.34	356.16 ± 1.83	320.58 ± 16.65 * ^c^	9.97 ± 5.12 ^c^	0.91 ± 0.05 ^c^
	1:45	7.2 ± 0.42	311.45 ± 9.45	375.99 ± 2.01	318.65 ± 9.87 ** ^c^	15.24 ± 3.07 ^d^	0.86 ± 0.04 ^bc^
Reducing power	1:70	6.98 ± 0.03	488.89 ± 2.23	1097.75 ± 20.43	495.87 ± 1.62 ** ^a^	54.82 ± 0.85 ^a^	0.45 ± 0.01 ^a^
	1:75	7.14 ± 0.02	534.95 ± 1.6	1144.18 ± 21.33	542.1 ± 1.62 ** ^b^	52.61 ± 0.81 ^b^	0.47 ± 0.01 ^b^
	1:80	7.97 ± 0.02	639.06 ± 2	1188.23 ± 22.19	647.05 ± 2.02 ** ^c^	45.53 ± 0.96 ^c^	0.54 ± 0.01 ^c^
	1:85	7.23 ± 0.02	614.13 ± 1.7	1230.08 ± 23.01	621.35 ± 1.72 ** ^d^	49.47 ± 1.09 ^d^	0.51 ± 0.01 ^d^

Note: EC_50Amix_ and EC_50Bmix_ indicate the EC_50_ values of antioxidants A and B in their combined solution respectively. EC_50mix_ is the experimental EC_50_ measured of the mixture of caffeic acid and SAC, and EC_50add_ is the theoretical EC_50_ of the mixture of caffeic acid and SAC. The letters indicate significant differences determined by Duncan’s multiple range tests (*p* < 0.05). * indicates significant difference when EC_50mix_ compared with EC_50add_ by independent *t*-test (*p* < 0.05), ** indicates extremely significant difference when EC_50mix_ compared with EC_50add_ by independent *t*-test (*p* < 0.01). It should be stated that the EC_50_ values are actually OD_0.5_ values in the part of reducing power in order to facilitate the presentation of table.

## Data Availability

All data generated or analyzed during this study are included in this article.

## Sample Availability

Samples of the compounds are available from the authors.
